# Facilitators and barriers for harm reduction after first use of novel nicotine delivery devices: a qualitative investigation of cigarette smokers

**DOI:** 10.1186/s40359-022-00874-w

**Published:** 2022-07-29

**Authors:** Gill Kinchen, Sharon Cox, Dimitra Kale, Lion Shahab

**Affiliations:** Department of Behavioural Science and Health, University College London, 1–19 Torrington Place, London, WC1E 6BT UK

**Keywords:** Novel nicotine delivery device (NNDD), Cigarettes/cigarette smoking (combustible), Heated tobacco product (HTP), E-cigarette

## Abstract

**Background:**

Novel nicotine delivery devices (NNDDs) are a safer alternative to combustible tobacco smoking. Understanding what factors can facilitate people who smoke to use NNDDs can inform intervention design and public health messaging. This study aims to explore the facilitators and barriers to NNDD use from the perspective of smokers without prior use, after trialling two NNDDs.

**Method:**

UK adults who smoke combustible cigarettes (n = 11) were recruited from a larger quantitative study after trialling two NNDDs, an electronic cigarette and a heated tobacco product (order randomly allocated). Semi-structured interviews were conducted, transcribed and thematically analysed, using the COM-B model of behaviour and NVivo12 pro software.

**Results:**

Five main themes were identified: health knowledge, availability of and accessibility to NNDD products, cost, social acceptance, and NNDD use experience. There was curiosity and interest in the uptake and use of NNDDs, but the absence of centralised product information was identified as a barrier. Other themes were related to the design and functionality of the NNDD products. For example, the e-cigarette with its low maintenance was seen as easier to use than the heated tobacco product, which offered too short a single use duration and was described as ‘cumbersome’. Throat discomfort and high anticipated cost were among additional barriers identified for both product types.

**Conclusion:**

This study highlights the need for reliable objective information on the health effects of NNDDs compared with combustible cigarettes, which could facilitate their regular use. Product adjustment such as single use duration alignment with combustible cigarette smoking duration may encourage uptake. Interventions offering opportunity for experience of NNDD use and knowledge dissemination of NNDDs could increase motivation to adopt harm reducing behaviours as demonstrated in this study.

**Supplementary Information:**

The online version contains supplementary material available at 10.1186/s40359-022-00874-w.

## Background

Smoking combustible cigarettes has a detrimental effect on health and leads to high demands being made on National Health Service resources in terms of the cost of investigations, treatments and staff involved in consequent care [1, 2]. Although the prevalence of cigarette smoking is at the lowest rate ever recorded at 14.4% in the UK (and is decreasing in many high-income countries worldwide), most quit attempts still result in failure with no more than 5% of attempts lasting one year [3]. High rates of relapse indicate how difficult people find it to achieve complete abstinence and it therefore makes sense to recognise the need for harm reduction measures [4]. Harm reduction can be defined as strategies designed to help people who smoke to reduce the harms from combustible tobacco, including gradual reduction in the number of cigarettes smoked over time, or by using an alternative nicotine delivery device (partial substitution), or fully switching over to such devices (complete substitution).

Many alternative systems of nicotine delivery exist, for example medicinal nicotine has been available for several decades and is the established recommended method for tobacco harm reduction in England [5]. Over the last decade there has been a proliferation of novel nicotine delivery devices (NNDDs), providing people who smoke with greater opportunity to use nicotine in much safer forms. Electronic cigarettes (e-cigarettes) became widely available in England from 2010, growing significantly in popularity amongst people who smoke and are now the most popular aid used for quitting [6]. Surveys by Action on Smoking and Health show that people in Great Britain use them primarily to quit or reduce smoking [7, 8]. Other harm reduction alternatives also exist, including heated-tobacco-products (HTPs), which heat tobacco rather than burning it, in doing so exposing the user to fewer toxicants and carcinogens [9, 10]. However, while many people try an e-cigarette, only around half go on to use them as a complete substitute for smoking, and there is a pressing need to develop the evidence on the facilitators and barriers to the use of NNDDs among current smokers. Recent qualitative research has investigated the health risk perceptions of NNDDs among current users but, not for current cigarette smokers, after trialling two NNDDs [11–13]. A preliminary data-base search revealed only one study which addressed a similar question in naïve users [14]. There is value, however, in understanding the facilitators and barriers to NNDD use among people who currently smoke and who are using the products in ‘real time’, as this provides an insight into the immediate and early-stage issues which people experience. It is also important to identify early facilitators to product use, so these can be maximised to help people switch sooner.

This exploration intends to address this gap in the literature using semi structured interviews. It forms part of a larger study investigating potential health effects of NNDDs by characterising their toxicological profiles. A subset of smokers were asked to trial NNDDs and were subsequently interviewed. The two main device types trialled in the current study are an e-cigarette and an HTP [9]. The main advantage of these devices is that there is no combustion involved in either and therefore fewer toxicants and carcinogens are produced. Both types of NNDDs have similar characteristics to cigarettes in addition to delivering nicotine, for example, requiring the hand to mouth movement and the act of inhalation and exhalation. The advantage of this method of nicotine delivery over other nicotine replacement therapies (e.g., patches) is that it retains some of the familiar characteristics of the person’s smoking habit [15]. This could make such NNDDs more likely to be adopted as harm reduction tools [8, 16].

The aims of the current study were: (1) to explore facilitators and barriers to using NNDDs for cigarette smokers after trialling two types of NNDD, and (2) to understand the findings in relation to the COM-B model and the Theoretical Domains Framework (TDF) [17–19].

The COM-B model of analysis enables the identification of the most influential factors impacting on a behaviour which can then lead to recognition of targets for change. This may be facilitators or removing barriers, which might most influence the modification of that behaviour over time. In mapping the domains of the TDF to this framework intervention design can be guided [18, 20].

## Method

### Study design

The current qualitative study interviewed a volunteering subset of smokers, participating in a larger study that aimed to investigate the health effects of NNDDs (see Additional file 1). Participants made two visits to the laboratory to trial two different types of NNDDs, an HTP product (the IQOS), and two e-cigarettes (the Juul (a pod device) and the Aspire PockeX (a refillable device)) delivering similar nicotine levels at 18 mg/ml or 2% respectively [15, 21, 22]. The flavours which participants could select from were tobacco or menthol and for the e-cigarettes a generic fruit flavour. This qualitative study used semi-structured interviews to capture individual perspectives on participants’ experiences of using NNDDs [23].

### Interview protocol

Semi-structured interviews were conducted over a two-month period (April–May 2019) and were held in a UCL laboratory. Interviews took place after each participant had trialled both types of NNDDs in alternating order to avoid order bias. Interviews were conducted by researchers HP and GK. Researchers maintained a professional demeanour to minimise any effects of interviewer bias. Prompts were used to support interviewer reflexivity; however, variations in participant readiness to expand and share influenced the breadth of individual responses. The reflexive process used by interviewers included prompts like ‘can you tell me a bit more about that?’ for relevant comments which might have elucidated a more in-depth reflection from the participant. This approach also served to clarify any points that were unclear. Currently a non-smoker with no strong view on harm reduction and no experience of NNDDs, but with an interest in health psychology, I feel this puts me in a position of relative neutrality as to the benefits of NNDDs and thus will have minimised bias in interpreting results.

### Topic guide

A semi-structured interview topic guide was developed by LS to cover views/experience of the products trialled. The question style was refined, adopting a more indirect type of question. For example, modifying ‘Why did you start smoking?’ to ‘Do you remember the situation in which you had your first cigarette?’ (see Additional file 5). Development was guided by the COREQ check list [24] and influenced by the COM-B model of behaviour, which provided the framework for analysis [25]. Probe questions were included to promote elaboration.

### Participants

A convenience sample of eleven smokers agreed to take part at the point of recruitment into the initial trial. Participants had to meet qualifying criteria: being over 18 years old; smoke a minimum of five cigarettes per day for a minimum of 6 months, and not ever being a regular user of any NNDDs (see Table 1). Although more participants would have been desirable, with nine interviewees meeting the saturation criteria for code saturation eleven participants was sufficient [26]. Written consent was obtained from all participants.

**Table 1:** Participant characteristics

Participant	Interview length minutes	Sex (M = male F = female)	Age	Ethnicity	Education level	Years smoking	NNDD trial order	
							1st visit	2nd visit
1	30	M	36	White British	Degree	16	E-cig	HTP
2	15	F	20	Asian Indian	Advanced/Level 3	2	E-cig	HTP
3	16	M	22	Asian Bangladeshi	Advanced/Level 3	5	E-cig	HTP
4	11	M	53	White British	Degree	37	E-cig	HTP
5	18	F	61	Asian Indian	Degree	31	HTP	E-cig
6	23	M	55	Mixed White/Asian	Advanced/Level3	37	HTP	E-cig
7	27	M	63	Mixed White/Black	Degree	38	E-cig	HTP
8	14	F	19	Other/white Turkish	High School	5	HTP	E-cig
9	25	M	40	White British	Standard/basic min education	22	E-cig	HTP
10	14	F	50	White British	Standard/basic min education	16	HTP	E-cig
11	9	F	61	White Irish	Degree	10	HTP	E-cig

*E-cig* electronic cigarette, *HTP* heated tobacco product

### Frameworks used in analysis

#### The COM-B framework

The COM-B framework has three component areas: capability (both psychological and physical), opportunity (social and physical), and motivation (reflective and automatic), which in varying combinations are considered essential to enable any behaviour to take place or be modified [17]. Operating as a dynamic system with feedback loops, the COM-B model allows the impact of components to be identified and targets for behaviour change to be developed ([25], see Additional files 2 and 3). When extended by mapping domains of the TDF to the COM-B framework interventions can be developed from theoretically based constructs, mainly relating to individual capability and motivation, in order to support and activate behaviour change [20].

#### The TDF framework

Of the 14 domains attributed to the TDF framework eight were identified as relevant to future intervention design for behaviour change in the current study. Presented in the order they first appear in Table 2 those domains were: 1. Knowledge; 6, Beliefs about consequences; 13, Emotion positive affect; 4, Beliefs about capabilities, self-efficacy; 12 Social influences, group norms and identities; 11 Environmental context; 9/10, Goals and intentions [18]. In columns three and four of the possible interventions table mapping between the frameworks can be understood.

### Data analysis

All interview recordings were securely stored and transcribed *verbatim* with transcriptions checked with the audio interviews and read multiple times to facilitate familiarisation. Then transcriptions were imported into NVivo12 pro to organise and manage the textual data [40], which was analysed using thematic analysis and inductively coded into coding categories using a reflexive process [41]. The COM-B framework was used to organise themes with each theme allocated to the most appropriate of the six COM-B sub-components.

Themes were identified by GK according to frequency of meaningful textual data, with a higher frequency considered to be a key theme. Revision of all themes was continual, and those with high frequencies of coded data were divided into separate categories. Codes with three or less items of data were not used but kept under a miscellaneous grouping. Short explanations of the effects of the themes on NNDD use were provided with textual data examples according to the COM-B model components.

### Analysis of contradictory results

Participants were encouraged to give their immediate impressions and opinions on their experiences of the devices trialled, all comments were welcomed and included in the analysis. Themes were developed in accordance with the comments made and if the same aspect was commented on negatively and positively and even neutrally, they were recorded under that aspect in the three different categories. It was perfectly acceptable for the same aspect of a theme to be found as a facilitator to use by one participant and a barrier to use by another. For example, in the Design theme regarding the Juul, P5 liked it because, “… it’s easy to put in my pocket.”, while P10 did not like it because, “I didn’t like how it felt in my hands…”. The frequency of comment types determined which themes became more important or key; an overwhelming number of negative comments on a particular aspect would have been presented as an outright barrier.

## Results

The convenience sample of cigarette smokers with a mean age of 43.6 years (SD = 16.4) was made up of six males and five females. Thematic analysis revealed 12 themes (four each in the psychological and physical capability component and one in each of the remaining four COM-B sub-components, see Additional file 2) of which five were considered key themes, i.e. were particularly prevalent: lack of health knowledge, ease of accessibility to products, initial cost of products with ongoing costs, social acceptability of NNDDs and motivation to use an NNDD, which was dependant on overall satisfaction with the trial product experience (see Additional files 3 and 4).

Among the 12 themes identified, the following were main barriers: health and product knowledge, which was seen as being inadequate by participants as they did not feel that they knew where to get such information; ongoing costs; harsh throat sensations; and single use durations. The main facilitators were social acceptability, cleanliness of products and a good trial use experience, and the remaining themes could be considered facilitators or barriers depending on participant perspective. Themes are presented in line with the COM-B model. (key themes with asterisks*; P = participant; F = facilitator; B = barrier).

### Psychological capability

#### Health knowledge*

The most common query arising from all participants was related to the lack of knowledge about the health effects of NNDD use, both short and long term, in comparison with cigarettes. Participants had a general awareness of the common health dangers of smoking but, in order to have confidence in investing psychologically and emotionally, as well as financially, in an NNDD for the purpose of reducing harm caused by cigarettes, participants felt that they needed convincing evidence of their comparative safety.

“I would like to know, kind of, about how it compares with standard cigarettes, and erm how much better it is for your health …” [E-cig] P11 F

“ ..also just even the look of it just feels dangerous because I think like, that liquid, I have no idea about what that is like, for example with cigarettes we know it’s a plant and it grows in the ground…. but that liquid seems very chemical….” [E-cig] P8 B

“I am kind of a fan of the IQOS I think. If it turned out to be, like, healthier then I would think about it seriously.” [HTP] P2 F

#### Understanding of harm reduction

Participants frequently displayed a lack of knowledge of the difference between nicotine and the carcinogenic toxicants found in combusted tobacco. Some feared that the use of NNDDs would lead to an additional addiction, by offering them a tool which they would tend to use in addition to their cigarettes rather than as a gradual replacement of them.

“I’m not too sure what I’m saying is right but, again if they contain nicotine, I can’t really see the health benefits of it. I don’t even know if they contain nicotine …” [E-cig] P6 B

“I don’t think it would help me stop smoking, I’d probably switch on to that and smoke a lot more. … I’d rather not have an additional problem.” [HTP] P9 B

“… there are a few flavours as well so … I think the range of flavours would kind of help kind of keep you off the nicotine kind of hit …” [HTP/E-cig] P3 F

#### Satisfaction, sensation and ritual

There were different levels of perceived smoking satisfaction provided by the products. Participants described throat and ‘high’ sensations in comparison to their normal cigarette smoking experience. The ‘high’ sensation description was explained as a light headedness. Descriptions of a similar sense of excitement in the pit of the stomach and of satisfaction at the thickness of the smoke as they inhaled from the HTP were reported. There were varying reports on throat sensations of harshness and throat irritation with all products. Some were happy about the new experience without the usual rituals associated with cigarettes, whereas others felt that the rituals were an essential part of their overall smoking enjoyment (feel of the paper when rolling a cigarette, flicking away of ash).

“… the very act of rolling it (a cigarette) is part of that ritual, … the feel of the paper, the filter on your lips … I don’t have any of that from the e-cigarette.” [E-cig] P1 B

“ … it’s actually easier to just whip it out and just use it. I think that for me personally, I’d find that quite beneficial in trying to stop.” [E-cig] P3 F

### Physical capability

#### Availability/accessibility*

It was suggested that easy access to and information about NNDDs are factors which would facilitate use. Participants did not feel that they knew where they could get comprehensive information from. The many intricate details that are part of the NNDD world were perceived, by some as overwhelming, hence presenting a barrier.

“… like, how much it costs? … I don’t’ know if you can get it in a normal shop … the ease of getting the product. … If you had to order online, and you couldn’t get it in time, then would you revert back to normal cigarettes?” [HTP] P10 F/B

“Is there any chance of doing try before you buy … if your spending money on buying the whole product … for people to try it, you may switch more people.” [HTP/E-cig] P4 F

#### Cost*

Most participants expressed the need to have an understanding of the cost benefit, beyond the initial outlay. There was a common expectation that cigarettes would still need to be purchased, at least initially, if and when participants began using an NNDD. Participants did not have enough knowledge to understand the fine distinctions between the potential ongoing costs of the individual products.

“… if it’s going to cost me more than my regular cigarettes of course I wouldn’t get it … the cost is a huge factor.” [E-cig] P5 F/B

“… I’d want to smoke tobacco as well or something … I don’t think they (NNDDs) would be cheaper financially.”” [E-cig] P9 F/B

“I feel like in order to buy a device and keep getting refills would be like a cost issue, especially as you can’t really switch between brands and stuff, …” [HTP] P2 B

#### Duration, design and functionality

The HTP was described as ‘cumbersome’ and the e-cigarette as easier to use in comparison. Having to be mindful of holding the button down detracted from enjoyment of the HTP and the unexpectedly short duration from satisfaction. The range of flavours was popular.

“… it was about half the time or less than that even of the time I’d usually spend having a cigarette … and I didn’t really realise it was finished until a while afterwards.” [HTP] P3 B

“I didn’t like how … it felt in my hands …” [HTP] P10 B

#### Maintenance

Participants expressed concerns about charging, needing new parts and one participant thought he would need a spare product in case he needed to wait for a spare part to be delivered. There was some anxiety around the prospect of the device not working and multiple sources of online information being confusing.

“I’d smoke a regular cigarette. If the tobacco refill was not available.” [HTP] P5 B

“ … so if you have a vape (E-cig) and the coil is broken or something and you think ah I’ll order a replacement part or whatever and then once you wait for that to come you can just go round the shops and then you just get back in the cycle again … I think if you had like a spare or a backup then I think it should be okay, …” [E-cig] P3 B/F

### Opportunity

#### Social acceptability*

There was a perception that using an NNDD would significantly reduce stigma associated with smoking. This perceived social acceptability was a strong facilitator for most participants. No longer smelling offensive to others was a source of satisfaction with NNDD use.

“… it's less, what's the word, reprehensible to the general public; … using the Juul could eliminate in many respects those negative reactions one does get when smoking a conventional cigarette.” [E-cig] P7 F

“… but, if someone saw it in my bag they wouldn’t know what it was, so that would make me feel a bit better about the product,…” [E-cig] P5 F

#### Physical convenience

There was more perceived tolerance towards NNDD users in indoor spaces, which can be helpful in supporting NNDD users. Some participants felt this lack of restriction could potentially encourage more intense use of NNDDs.

“I’m working and I need to get out of the office, to smoke a full cigarette, it takes that much longer … easier to just go out and have a couple of drags of an e-cigarette …” [E-cig] P5 F

### Reflective motivation

#### Boost and new confidence*

Positive experiences of the products increased motivation. Participants remarked on their surprise at the number of NNDDs available and reported feeling encouraged by this knowledge.

“I think my opinion has changed dramatically since last week … – it’s been such a good experience …. that’s the first time I’ve even considered it (quitting cigarette smoking) in 30 years so that says something for the product.” [E-cig] P5 F

“… there’s a lot more out there than I knew there was. I just thought there were e-cigarettes, …” [HTP/E-cig] P10 F

“… I think actually trying it just does kind of help me personally to realise … I’m a bit more determined because I’ve kinda seen what’s out there …” [HTP/E-cig] P3 F

### Automatic motivation

#### Desire to continue smoking

In contradiction to the enthusiasm shown by some, the intention to continue cigarette use arose in participants who considered that there was currently no health threat posed by their smoking habit and therefore any suggestions around quitting were redundant.

“But I don’t really have any particular desire to, (quit) as I say, because I’m smoking so little and I’m otherwise healthy …” [HTP/E-cig] P1 B

### Association of sociodemographic characteristics with themes

There were double the number of negative comments made about the e-cigarettes by male participants, mainly under the themes of Satisfaction, sensation and ritual (29%), mentioned by five of the six male participants, followed by Physical convenience in its negatively perceived aspect of less limitation on use, and Desire to continue smoking (both 18%). Next was the theme of Understanding of harm reduction (12%), and finally at a lower frequency under the themes of Health knowledge, Cost, Duration design and functionality, and Maintenance (each at 6%). Otherwise there appeared to be no trends which differentiated between participants’ age, sex or ethnicity. There was a higher frequency of comments from participants who had reached a higher educational attainment.

### Implications for behaviour modification interventions

The following description of results outlining facilitators and barriers to NNDD use indicates specific areas as suitable targets for potentially effective interventions.

A lack of reliable health effect information of NNDDs and harm misperceptions due to a lack of knowledge were barriers for participants. Unfamiliarity with product information lead participants to indicate that they were curious to learn more about NNDDs in order to aid decision making and cost queries were expressed as a common concern, presenting further barriers to use. There were mixed views on participant satisfaction with the ritual and experience of the products. Participants tended to favour one or other of the device types (e-cigarette or HTP) for its aesthetic and/or functional appeal. Reports indicate that the satisfactory single use experience of the HTP was comparable to cigarette smoking and would facilitate product use. However, the HTP single use duration was reported as being too short, participants felt that this disparity would lead to increased use, by the desire to use a second tobacco stick. Participants enjoyed the mouth tip similarity to cigarettes, the flavour selection and there were varying positive reports of the light-headedness experienced. Barriers to HTP use included bulkiness of the product, complication of loading tobacco sticks, having to hold a button down and charging after every use.

The e-cigarettes were more popular, due to their cleanliness, convenience, simplicity of use and once per day charge time. Flavours were a facilitator although, there were negative reports on the e-cigarette use experience not ‘feeling’ like smoking and the expectation that the unlimited duration would lead to increased use, both presenting barriers to uptake. Some participants expressed relief at the prospect of using an NNDD as this would be more aligned with their desired potential ‘non-smoker’ identity. Participant motivation to take up NNDD use was increased simply by exposure to knowledge of the existence of the harm reducing products. The level of satisfaction of the participant experience of the NNDD trial fed into their motivation and perceived capability to use an NNDD. For others, the desire to continue smoking and the perceived lack of a direct health risk remained barriers to NNDD use.

Implications in relation to facilitators and barriers derived from the themes are summarised in Table 2 with suggestions on how to address these through interventions, based on the COM-B and TDF frameworks (see Table 2).

**Table 2:** Identified facilitators and barriers and possible interventions informed by the literature, COM-B [17] and TDF [18]

COM-B sub-components		Facilitators and barriers identified	TDF	Intervention function	Possible intervention
1. Psychological capability	a*	Health knowledge confusion over harm-reduction and need to have factual evidence that NNDDs are safer than cigarettes	Knowledge and beliefs about consequences	Increase availability of knowledge of comparative health implications	‘Try before you buy’ outlet with face to face trained staff
				Disseminate evidence of harm reduction	Explanatory email/leaflet/library/TV advert on harm reduction [27–29]
	b	Satisfaction	Emotion (positive affect)	Trial to experience sensations	Hygiene safe trial and report site [30]
		Good sensations similar to cigarette smoking			
2. Physical capability	a*	Cost	Beliefs about capabilities	Make current cost details available to enable goal planning	Helpline—email link. A link with monthly updated information since prices are going downward [17, 31, 32]
	b*	Availability/access	Self confidence/self efficacy	Easy access to reduce self-consciousness	Universal retail outlet—High St. easy access; trained staff [25, 33, 34]
	c	Duration Need provision to increase length of smoking time for the HTP Include a limitation on E-cigs		Modification of design	Longer tobacco sticks for HTP trial and time limiter on E-cigs [35]
3. Opportunity social	a*	Social acceptability	Social influences (norms and conformity)	To reinforce *idea of being with the in crowd??* Modelling	Self-experienced
		Encouragement as self-perception improves—no bad smell. Others moving on to this form of NNDD	Social/professional role and identity	Progressing toward perceived desired identity	Share experiences
					Desired non-smoker identity [19, 36]
4. Opportunity physical	a	Convenience Possibility of short period using an NNDD for a specific number of puffs (E-cigs)	Environmental context and resources	Modelling convenience use—short burst of NNDD nearby as allowed. Disgruntled cigarette smoker returning from a lesser further away place	Separate NNDD users from cigarette smokers and have some nicer place for NNDD users [18, 37]
5. Motivation reflective	a*	Renewed optimism Simply by coming into contact with the products. Learning of their existence	Goals and Intentions (implementation intention/intrinsic motivation)	Facilitate experience Hear about others’ experience	More information points for guidance for cigarette smokers to trial such products [21]
6. Motivation automatic	a	Intention to continue smoking cigarettes	Beliefs about consequences	Negative health consequence feedback	Education and information sharing of health consequences
		Health derogation	Absence of social influence to change behaviour	New interest; social expansion—identity modelling	Promotion of an interest combining social-interaction [38, 39]

*Key themes

## Discussion

Findings from this exploration of facilitators and barriers for harm reduction in smokers identified five key themes determined by highest to lowest frequency of meaningful textual data: (1) health-knowledge, (2) availability of and accessibility to NNDDs, (3) cost of NNDDs, (4) social acceptability of NNDD use and (5) increased motivation to use NNDDs as a harm reduction measure. These results concur with recent research [11, 12].

### Health-knowledge and risk perceptions

The perception of susceptibility to negative effects of cigarettes combined with perceived benefits of harm reduction, can affect enactment of health promoting action (Health Belief Model), [13]. Perceived barriers upheld by low health literacy can obstruct any cue that might trigger this process [42]. Harm perceptions were confused by a lack of knowledge of the comparative harm of products trialled and cigarettes. Research has shown risk misperceptions of potential harm from nicotine and other carcinogens experienced by both people who use NNDDs and people who smoke [27]. Different attempts have been made using framing strategies to impart correct health information, finding that participants have difficulty applying knowledge when assessing risk [28, 30]. The dissemination of more accurate health risk information could be addressed, for example, by using ‘fun’ online multiple-choice quizzes on an NHS patient portal, or with direct and anonymous access.

### Availability and accessibility to NNDDs

Although products are available in retail outlets, potential users also need appropriate and correct NNDD information at the same outlet in order to encourage NNDD use [43, 44]. For potential users to believe that they have control over the enactment of a behaviour (NNDD use) they need to have good access to the broad spectrum of NNDD products facilitating perceived behavioural control (Theory of Planned Behaviour), [45]. The sense from participants was that there needed to be a central retail NNDD ‘supermarket’ where participants could see products and related components and find answers to their questions as they arise, from well-trained, knowledgeable advisors in a ‘face to face’ situation. This could allay any feelings of self-consciousness from going into a small retail outlet offering limited stock and thereby facilitate NNDD uptake and use.

### Cost uncertainties

In such a comprehensive retail outlet advisors could assist potential users by calculating individual expected expenditure. Participant comments expressed a common concern regarding the affordability of NNDD use uptake citing it as a potential barrier.

### Social acceptability

In contrast, the popularity of the improved cleanliness of NNDD use, avoiding unpleasant smells, was a strong facilitator, leading people to feel better about their product use. The Identity Shift Theory claims that practicing a new behaviour can lead to small changes in participant attitude, moving towards behaviour change and eventually identity change [36, 46, 47]. A strategy to support this gradual identity shift could be to provide pleasant areas for using NNDDs within the workplace, this could motivate smokers to switch to NNDD use and evoke a sense of belonging to a less ostracised group.

### Motivation

Participants’ motivation to take up NNDD use was increased simply by exposure to knowledge of the existence of the harm reducing products. The emotional response generated by some participant experiences increased motivational power in line with the PRIME theory (plans, responses, impulses, motives and evaluations), which leads momentary desire to inspire behaviour modification plans [48]. If a ‘try before you buy’ facility could be offered and possibly subsidised by product manufacturers, this would eliminate a number of barriers identified by participants. This could offer the opportunity for a trial experience in a relaxed and welcoming environment which could increase motivation for NNDD use uptake.

### Automatic motivation

A further barrier to harm reduction in continuing smokers was the derogation of health risk susceptibility [49]. This cognitive disparity between awareness of the health risks posed by cigarette smoking and the continuation of the risk behaviour has been theorized as cognitive dissonance [50] and fits into the automatic motivation component of the COM-B model.

### Other observations

The fact that twice as many males criticised the e-cigarette may be indicative of sex preferences for certain features, and this would be interesting to investigate further with a larger representative sample.

The main limitation of this study was the non-representative convenience sample and small number of participants. The main advantages were the focus on current smokers naïve to NNDD use, highlighting immediate barriers and facilitators and the mapping of the COM-B components on to the TDF enabling intervention suggestions.

## Conclusion

In conclusion, this study identified five facilitators and barriers which could be the focus of interventions, with an outstanding need for information on the health effects of NNDDs. Facilitators included comprehensive product accessibility, cost information, social acceptance and the effect of NNDD experience on motivation. It also identified the main barriers as being a lack of knowledge in multiple areas and a lack of familiarity with the products. The modification of the single use duration for both products, aligning it with cigarette smoking time, may eliminate this barrier to NNDD enjoyment. This study has demonstrated that motivation to reduce harm can increase with knowledge and experience of NNDDs, suggesting great scope for information and experience disseminating interventions.

## Supplementary Information

**Additional file 1.** Flowchart showing quantitative study procedure outline with current qualitative study interviews.

**Additional file 2.** Mind-map of COM-B model themes and development.

**Additional file 3.** Table of results showing facilitators and barriers from participants’ comments and presented using the COM-B framework.

**Additional file 4.** Bar chart showing key theme response frequencies.

**Additional file 5.** Topic guide.

## Data Availability

Availability of anonymised interview transcripts: https://osf.io/5npgv/?view_only=ccaa42634f1644fca18dbea61959c7e6.

## Abbreviations

COM-B: Capability, opportunity and motivation model of behaviour
E-cig: Electronic cigarette
HTP: Heated tobacco product
E-cig/HTP±: E-cigarette/HTP positive/negative response
F/B: Facilitator/barrier
MotA/MotR: Automatic motivation/reflective motivation
NNDD: Novel nicotine delivery device
P: Participant
TDF: Theoretical domains framework
