# Supplementary material for: Facilitators and barriers for harm reduction after first use of novel nicotine delivery devices: a qualitative investigation of cigarette smokers
Source: BMC Psychol. 2022 Jul 29;10:190. doi: 10.1186/s40359-022-00874-w (PMC9336076; doi:10.1186/s40359-022-00874-w)
Supplement: Supplementary file 1 — Additional file 1. Flowchart showing quantitative study procedure outline with current qualitative study interviews. [file 40359_2022_874_MOESM1_ESM.docx]

Facilitators and Barriers for NNDD use Supplementary files

**Facilitators and barriers for harm reduction after first use of novel nicotine delivery devices: a qualitative investigation of cigarette smokers.**

**Additional file 1.** Flowchart showing quantitative study procedure outline with

**
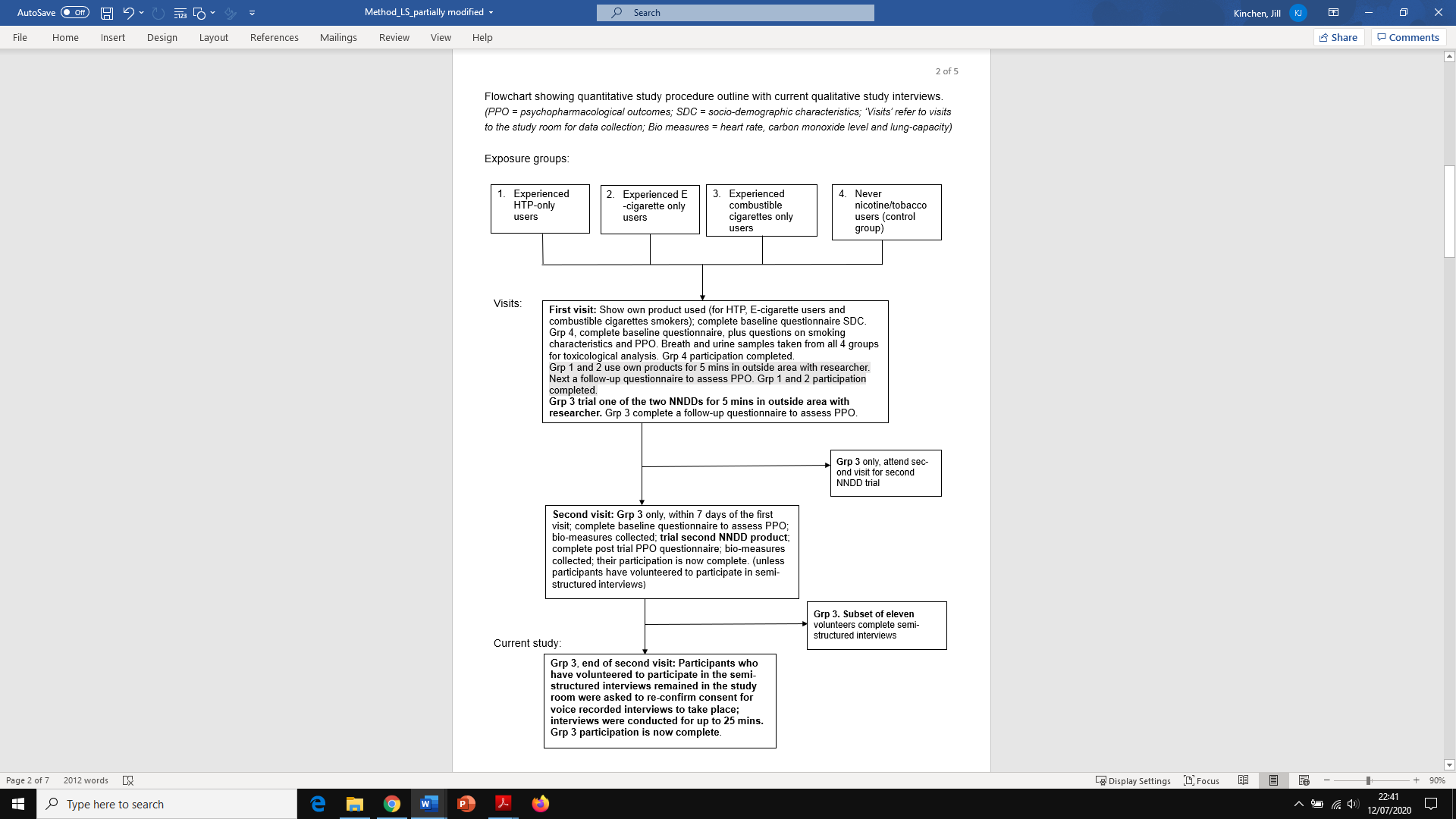
**current qualitative study interviews.

*(PPO=psychopharmacological outcomes; SDC=socio-demographic characteristics; ‘Visits’ refer to visits to the study room for data collection; Bio measures=heartrate, carbon monoxide level and lung-capacity)*
