# Supplementary material for: Facilitators and barriers for harm reduction after first use of novel nicotine delivery devices: a qualitative investigation of cigarette smokers
Source: BMC Psychol. 2022 Jul 29;10:190. doi: 10.1186/s40359-022-00874-w (PMC9336076; doi:10.1186/s40359-022-00874-w)
Supplement: Supplementary file 2 — Additional file 2. Mind-map of COM-B model themes and development. [file 40359_2022_874_MOESM2_ESM.docx]

**Additional file 2**. Mind-map of COM-B model themes and development

PSYCHOLOGICAL

Harm Reduction

**CAPABILITY**

Health Knowledge*

Design

PHYSICAL

**Satisfaction**

**OPPORTUNITY**

Cost*

Maintenance

SOCIAL

PHYSICAL

Availability & Accessibility*

Social Acceptability*

Increase in smoking locations

**MOTIVATION**

REFLECTIVE

Positive experiences increasing motivation*

AUTOMATIC

Remaining a smoker

In CAPABILITY – code **Satisfaction** developed into two types of satisfaction: Psychological and Physical.

**CAPABILITY**

PSYCHOLOGICAL

Harm Reduction

Cost*

Health Knowledge*

PHYSICAL

Design

**Satisfaction**

Duration & Functionality

Maintenance

**Satisfaction**

Sensation & Ritual

Availability & Accessibility*
