# Supplementary material for: Facilitators and barriers for harm reduction after first use of novel nicotine delivery devices: a qualitative investigation of cigarette smokers
Source: BMC Psychol. 2022 Jul 29;10:190. doi: 10.1186/s40359-022-00874-w (PMC9336076; doi:10.1186/s40359-022-00874-w)
Supplement: Supplementary file 3 — Additional file 3. Table of results showing facilitators and barriers from participants’ comments and presented using the COM-B framework. [file 40359_2022_874_MOESM3_ESM.docx]

**Additional file 3.** Table of results showing Facilitators and Barriers from participants’

comments and presented using the COM-B framework

| Themes and sub-themes | **Facilitators** | | **Barriers** | |
| --- | --- | --- | --- | --- |
|  | HTP/IQOS | E-cigs/Juul & Aspire | HTP/IQOS | E-cigs/  Juul & Aspire |
|  | ***Psychological capability*** | | | |
| Health knowledge*  Understanding of harm reduction | - Participants needed facts about health effects of both products compared to cigarettes. - Details on what constitutes harm reduction. | | - Lack of health knowledge is a barrier to confidence to use NNDDs. | |
| Satisfaction, sensation and ritual | - Similar sensations and smoking experience - Absence of time-consuming ritual involved in rolling one’s own cigarettes - Thicker smoke – sensed at inhale, similar to cigarettes - Light headedness or ‘buzz’ – comparable to cigarettes | - Absence of time-consuming ritual - Good taste/flavour - Small and easy to put into pocket - Smooth and   satisfying | - Absence of ritual enjoyment - Harshness on throat | - Absence of ritual enjoyment - Some throat and taste unpleasantness - Not strong enough |
| Design | - Similarities to cigarettes -mouth tip HTP/IQOS - Familiar shape (cylindrical like cigarettes) | - Aesthetically pleasing E-cig/Juul design - Small and   pocketable | - Button to hold down - Charge after each use - Awkward to hold - Heavy | - Dislike of E-cig/Juul square design - Awkward to hold |
|  | ***Physical capability*** | | | |
| Availability/  access* | - Need for access to products and product information | | - Unsure where to buy product | - Unsure about   product  details |
| Cost* | - Hoped costs would be comparable to cigarette habit - Tailored cost details would be a facilitator | | - Hidden cost of maintenance | - Unsure about   cost details of components |
| Duration and satisfaction | - HTP/IQOS Good light-headedness and similar smoking sensation - Cleaner habit - Flavours attractive | - E-cigs convenient for short time use when appropriate (eg; working day) - Cleaner habit - Flavours attractive | - HTP/IQOS too short a duration - Button to hold down - Too much smoke | - E-cigs - No time-limit – tendency to smoke for too long - Not very strong |
| Maintenance | - Need for information about parts that may need replacement over time | - Only one charge per day - Pod replacement/   refill simple | - Constant charging necessary throughout the day - Expensive - Anxiety about breakdown (non-universal parts) | - Lack of   Understanding of maintenance  necessary  (e-liquid;  Coil replacement) |
| **Opportunity** |  |  |  |  |
| Social acceptability* | - HTP/IQOS and E-cigs welcome social approval | | - Could facilitate   increased use due to less  apparent disapproval | |
| Physical convenience | - Use of both product types usually catered for in multiple locations (unlike Cigarettes) | | - Could facilitate increased use due to   use acceptance  in multiple locations | |
|  |  | - E-cigs Few puffs possible at one time |  |  |
| **Motivation** |  |  |  |  |
| Reflective* | - Discovery of new NNDD which would be interesting to try - Satisfactory smoking experience | - Boost with new confidence after use of NNDD and good experience - Smooth smoking experience | - Might be too difficult to maintain - Might be too expensive | - Might be too expensive |
|  |  |  |  |  |
|  |  |  |  |  |
| Automatic |  |  | No intention to quit cigarettes – no health issues | |
| **Key themes with asterisk; ‘cigarettes’ refers to combustible cigarettes* | | | | |
