# Supplementary material for: Facilitators and barriers for harm reduction after first use of novel nicotine delivery devices: a qualitative investigation of cigarette smokers
Source: BMC Psychol. 2022 Jul 29;10:190. doi: 10.1186/s40359-022-00874-w (PMC9336076; doi:10.1186/s40359-022-00874-w)
Supplement: Supplementary file 4 — Additional file 4. Bar chart showing key theme response frequencies. [file 40359_2022_874_MOESM4_ESM.docx]

**Additional file 4.** Bar chart showing key theme response frequencies
