# Supplementary material for: Facilitators and barriers for harm reduction after first use of novel nicotine delivery devices: a qualitative investigation of cigarette smokers
Source: BMC Psychol. 2022 Jul 29;10:190. doi: 10.1186/s40359-022-00874-w (PMC9336076; doi:10.1186/s40359-022-00874-w)
Supplement: Supplementary file 5 — Additional file 5. Topic guide. [file 40359_2022_874_MOESM5_ESM.docx]

**Additional file 5.** Topic guide

| **Objective:** To explore what factors would facilitate use of e-cigarettes and heat-not-burn/HTPs (heated tobacco products) and discourage conventional cigarette smoking. | |
| --- | --- |
| **Interview topics/questions** | **Probes** |
| Smoking history  *Question: Can you tell me a bit about yourself and how you came to start smoking?* | No more probes here (covered in quantitative)   - Do you remember the situation in which you had your first cigarette? |
| Quit attempts  *Question: Have you ever attempted to quit smoking?* | *If attempted to quit:*   - Could you tell me about your most recent quit attempt? - What made you give up on your quit attempt(s)/why do you think your quit attempt did not last? - What is your attitude towards smoking now?   *If no quit attempts:*   - Why do you think that is? - Could you tell me whether you plan to quit now / in the future? |
| Alternative nicotine/tobacco products  *Question: Can you tell me whether you have ever tried any alternative nicotine/tobacco products in the past* ***as an aid to quit smoking*** *(e.g. nicotine gum, patch, lozenge, e-cigarette, and heat-not-burn?)* | *If yes:*   - Can you tell me about your experience of using this/these product(s)? - Why did you try that product? - Was there anyone you spoke to about choosing to use this product? - What did you think about the product? - For how long did you use it? - Why did you stop using it?   If no: next question. |
| Views/experience of products tried in study (EC/HNB)  *So you’ve now tried both an e-cigarette (JUUL) and a heat-not-burn device (IQOS).*  *Question: Can you tell me what you think about the JUUL* ***as an aid to quit smoking****?*  *Questions: Can you tell me what you think about the IQOS* ***as an aid to quit smoking****?* | *Ask for both products:*   - How familiar were you with this product before taking part in the study?   (Have you read/heard anything about this product before? If so, from whom?)   - How does it compare to smoking? E.g. taste/smell/appearance? - What would you say are the potential benefits to you of using this product? - What would you say are the potential downfalls to you of using this product? - How do you think this product could help you quit smoking? - What do you think this product should be used for (e.g. harm reduction/cutting down smoking/quitting smoking/hobby?) - How would you feel about using this product again in the future? - What, if any, information or support would you like to receive about using this product?   *If don’t want to use the product again:*   - What would have to change in order for you to use this product in the future? |
| Ending the interview  *Question: Is there anything else you would like to add?* | - Do you have any questions regarding the research? - If you know any smokers who would be interested in participating in the study, please pass on our details (*provide flyer*). - *Provide participant with researcher’s contact details, should anything else come up* - Thank the participant |
